# In vitro gastrointestinal digestion of *Lentinus squarrosulus* powder and impact on human fecal microbiota

**DOI:** 10.1038/s41598-022-06648-z

**Published:** 2022-02-16

**Authors:** Francis Ayimbila, Supatcharee Siriwong, Massalin Nakphaichit, Suttipun Keawsompong

**Affiliations:** 1 Specialized Research Units: Prebiotics and Probiotics for Health, Department of Biotechnology, Faculty of Agro-Industry, Kasetsart University, Bangkok, 10900 Thailand; 2 Center for Advanced Studies for Agriculture and Food, KU Institute of Advanced Studies, Kasetsart University (CASAF, NRU-KU), Bangkok, 10900 Thailand; 3 Synchrotron Light Research Institute (Public Organization), Nakhon Ratchasima, 30000 Thailand

**Keywords:** Biological techniques, Biotechnology, Microbiology

## Abstract

Humans have long-used mushrooms as food and medicine, but digestion and colonic fermentation of most mushrooms, including *Lentinus squarrosulus* is markedly unknown. Here, nutritional profile, digestion and colonic fermentation of *L. squarrosulus* powder (LP) were determined. The powder contained mainly carbohydrate and protein. SEM and F-TIR analysis of the resistant hydrolysate (RH) revealed that the structure and ratio of carbohydrate and protein components were altered, and released known immunomodulation agents; beta-glucans and mannose. Both LP and RH promoted selected probiotic bacteria, especially *Bifidobacterium* strains. Using fecal microbiota of five volunteers (V1, V2, V3, V4 and V5), RH stimulated the microbiota of all used volunteers, via decreasing the ratio of *Firmicutes/Bacteroidetes* ranging from 1.3 to 8.2 times. Also, RH increased the relative abundance of vital immunomodulators; *Bacteroides*, *Bifidobacterium*, *Clostridium* cluster XIVa and IV, and *Sutterella*. Additionally, RH fermentation enriched the content of branch-chain fatty acids (BCFA) and short-chain fatty acids (SCFA), indicating protein and carbohydrate usage. Notably, propionic and butyric acids were abundant in V1, V2 and V3, while in V4 and V5, acetic and butyric acids were most enriched. Suggesting *L. squarrosulus* as functional mushroom to improve health and prevent diseases by enhancing gut health.

## Introduction

The gastrointestinal (GI) tract play important role in human health^1^. It has been shown that food matrix determines food stability during gastric and intestinal digestion, and the proportion that reach the colon or that will potentially be absorbed^2^. Nowadays, concerns about GI health problems including inflammatory bowel disease (IBD)^3^, diabetes^4^, cancer^5^, and obesity^6^ and liver disease^7^ have increased. Meanwhile, functional food components as potential therapeutic agents are being highlighted^8^. Edible mushrooms are rich in functional ingredients, and have since been consumed and are valued for their low calorific value, and rich compounds such as carbohydrate and protein that are indigestible but can be utilized by gut microbiota^9^.

The human microbiota is dynamic and complex, and its composition is shown to differ widely across healthy individuals. The gut microbes co-evolve with individuals that provide the microbes with stable environment, while the microbes participate in a series of metabolism including digestion and fermentation of food^10^. Variation in the microbiota among individuals takes place in response to diseases and environmental factors^11^, whereas substantial changes have been documented in response to mushroom active compounds^12^. Previous reports have indicated that different edible mushrooms and their functional components affect host intestinal flora and mechanisms, prospect to improve health and prevent diseases. Edible mushrooms (*Auricularia auricular, Flammulina velutipes, Lentinus edodes, Agaricus bispours, Pleurotus osteratus* and *Pleurotus eryngii*) were reported to have a positive role in gastrointestinal tract health by producing SCFAs and regulating the intestinal microbiota^12^. Chang et al.^13^ found that *Ganoderma lucidum* polysaccharides produced microbiota-modulating effects and could be used as prebiotics agents to prevent obesity. While it is understood that mushrooms contain mainly carbohydrate and protein, which may synergistically affect microbiome composition, however, most reports focused on the impact of the carbohydrate fraction but not the protein. Since, people generally consumed whole mushroom^14^, it is important to understand the effect of gastrointestinal condition on whole mushroom matrix and the impact of the indigestible fraction on human gut microbiota.

*Lentinus squarrosulus* is an edible mushroom belonging to Polyporales, which is mostly consumed in Thailand, and other parts of Asia and central Africa^15^. This mushroom has recently been highlighted due to a wide range of bioactive properties including immunomodulation^16^, antimicrobial^17^, antiproliferative^18^ and antiulcer^19^ studied in vitro. Our recent report indicated that *L. squarrosulus* contains various polysaccharides with medicinal value^20^. Obviously, due to the rich source of functional components such as carbohydrate, dietary fiber, protein and low fat^19^. Notably, most of the biological activities of *L. squarrosulus* are linked to its carbohydrate and protein fractions. Despite the fact that many of the biological properties of this mushroom have been reported, there is no information about its digestion and colonic fermentation. Carbohydrate and protein metabolisms were considered as markers for the study. Therefore, we evaluated simulated digestion profile of *L. squarrosulu*s powder, and the effect of its resistant hydrolysate on the fecal microbiota of different individuals. Specifically, our findings outlined the comprehensive and beneficial effects of *L. squarrosulus* on human gut health.

## Results and discussion

### Nutritional value analysis of *L. squarrosulus* powder (LP)

The results of the nutritional value (g/100 g) obtained for *L. squarrosulus* are presented in Table 1. LP contained 55.50 g carbohydrate, 30.12 g protein, 2.29 g fat, 5.27 g moisture, 94.73 g dry matter (DM), 6.82 g ash and 18.25 nitrogen free extract (NFE). The carbohydrate value was comparable to that of some medicinally valued species of *Pleurotus*; *P. flabellatus* (57.4%) and *P. florida* (63.0%) (Raman et al., 2021), but higher than that of *Lentinula edodes* (30.2%). Also, protein content of *L. squarrosulus* was higher than that of *Lentinula edodes* (17.0%), but similar to the amount reported in *P. citrinopileatus* (30.0%) and *P. djamor var. roseus* (35.5%) (Raman et al., 2021). *L. squarrosulus* exhibited fat content slightly higher than *L. edodes* (1.9%)^21^. Carbohydrate was the major component in *L. squarrosulus*, and crude fiber fraction was 14.81 g. This was equivalent to oyster mushrooms, which range from 11.2 to 15.0%^22^, and was relatively low compared with *L. edodes* (39.4%)^21^. The detergent fiber profile was analysis, which consisted of 37.26 g neutral detergent fiber (NDF), 15.62 acid detergent fiber (ADF) and 1.03 g acid detergent lignin (ADL). Cellulose content was 14.59 g and the value of hemicellulose was found to be 21.65 g. The concentration of β-(1,3–1,6)-glucan and α-glucan were 29.49 g and 0.55 g, respectively. Lastly, the energy value was 349.315 kcal/100 g dw or 19.5 kcal/100 g fw, which is lower compare to *Agaricus bisporus* (~ 29 kcal/100 g fw), *Pleurotus* spp. (~ 39 kcal/100 g fw), *Lentinula edodes* (~ 73 kcal/100 g fw) and *Flammulina velutipes* (~ 43 kcal/100 g fw)^23^. The results indicated that LP consist of essential carbohydrates and substantial amount of protein, and low fat, which can be consider as a low-calorie food. Also, the high fiber composition may greatly influence it digestion and impact on gut microbiota of human.

**Table 1:** Physico-chemical composition of LP (g/100 g DM, mean ± SD).

Composition	Value
Moisture	5.27 ± 0.12
Dry matter (DM)	94.73 ± 1.63
Ash	6.82 ± 0.26
Carbohydrates	55.50 ± 0.87
Fat	2.29 ± 0.11
Protein (% N × 6.25)	30.12 ± 0.74
Nitrogen free extract (NFE)	18.25 ± 0.56
β-(1,3–1,6)-glucan	29.49 ± 0.07
α-glucan	0.55 ± 0.012
Neutral detergent fiber (NDF)	37.26 ± 0.22
Acid Detergent Fiber (ADF)	15.62 ± 0.36
Acid Detergent Lignin (ADL)	1.03 ± 0.03
Cellulose	14.59 ± 0.40
Hemicellulose	21.65 ± 0.46
Crude fiber	14.81 ± 0.58

### Simulated gastro-intestinal (GI) digestion of LP

#### Morphological changes during digestion

**Figure 1:**
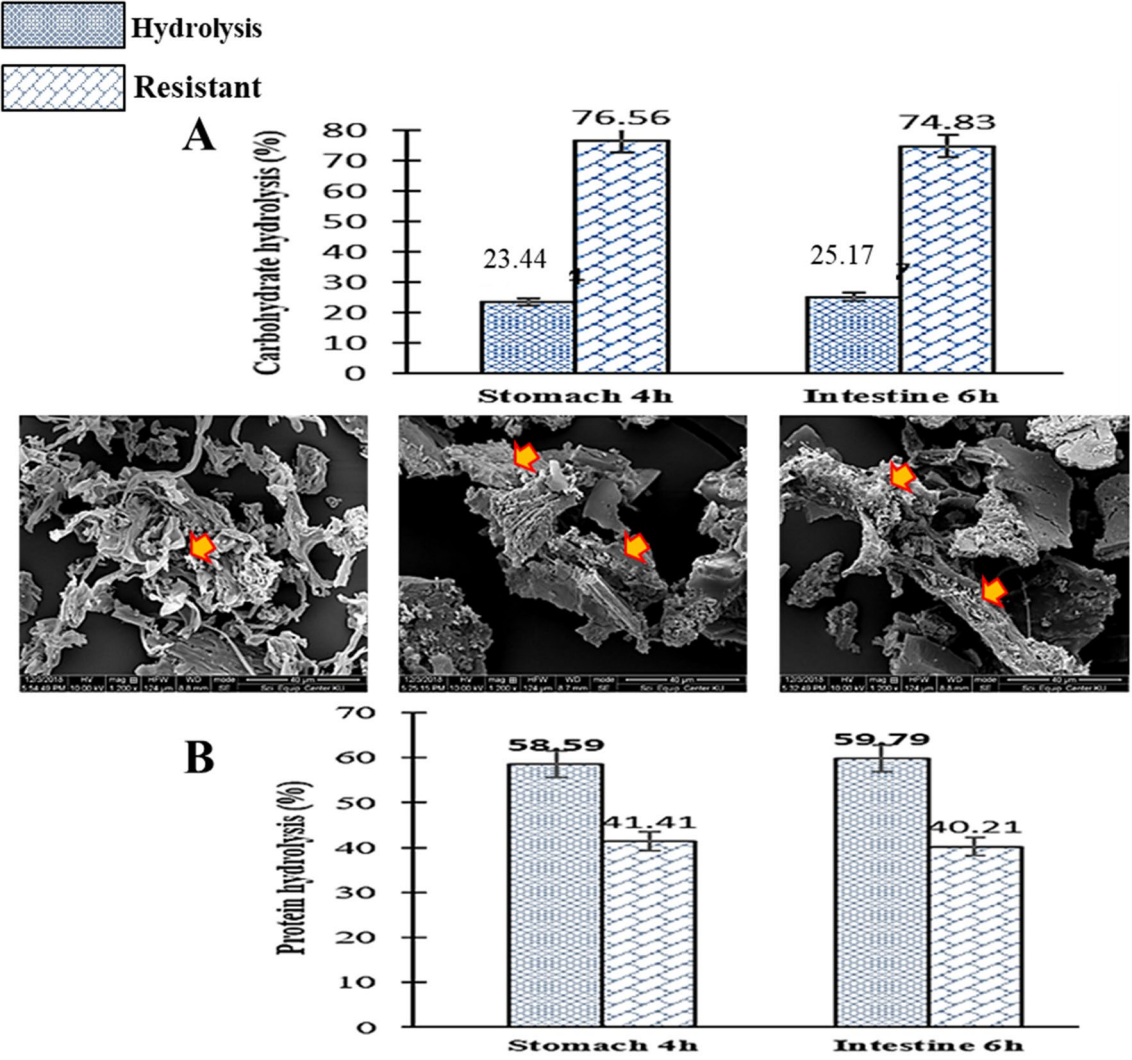
Scanning electron microscope (SEM) images, and the percentages (%) rate of carbohydrate (**A**) and protein (**B**) digestion in simulated GI condition. Before digestion (0 h), gastric/stomach digestion (4 h, pH 1, pepsin) and intestinal digestion (6 h, pH 6.9, pancreatic α- amylase).

During digestion, the structure of food particles are altered due to disruption of aggregates and breakdown of linkages^24^. Therefore, SEM image analysis was used to observe the effect of simulated GI condition on the hydrolysate of LP particles. As evidenced by SEM images in Fig. 1, before (0 h) digestion, the structures appeared compact and the cell shape maintained connective-like structures, with smooth surfaces. After digestion in gastric juice for 4 h, LP micro-particles looked degraded and shorten, with holes on some of the surfaces. Subsequently, a little more structural changes were observed after digestion in intestinal fluid, especially development of holes on some of the surfaces. The degradations and shortening of particle structures can be related to separation of aggregates and breakdown of linkages due the homogenization and low pH during digestion. Also, the holes on the surfaces of some of the particles can be attributed to the activities of pepsin and porcine pancreatic α- amylase^24^. Interestingly, most of the particles retained their shapes, possibly as a result of resistant to the low pH and the enzymes activities.

#### Structural changes altered nutritional profile of LP

**Figure 2:**
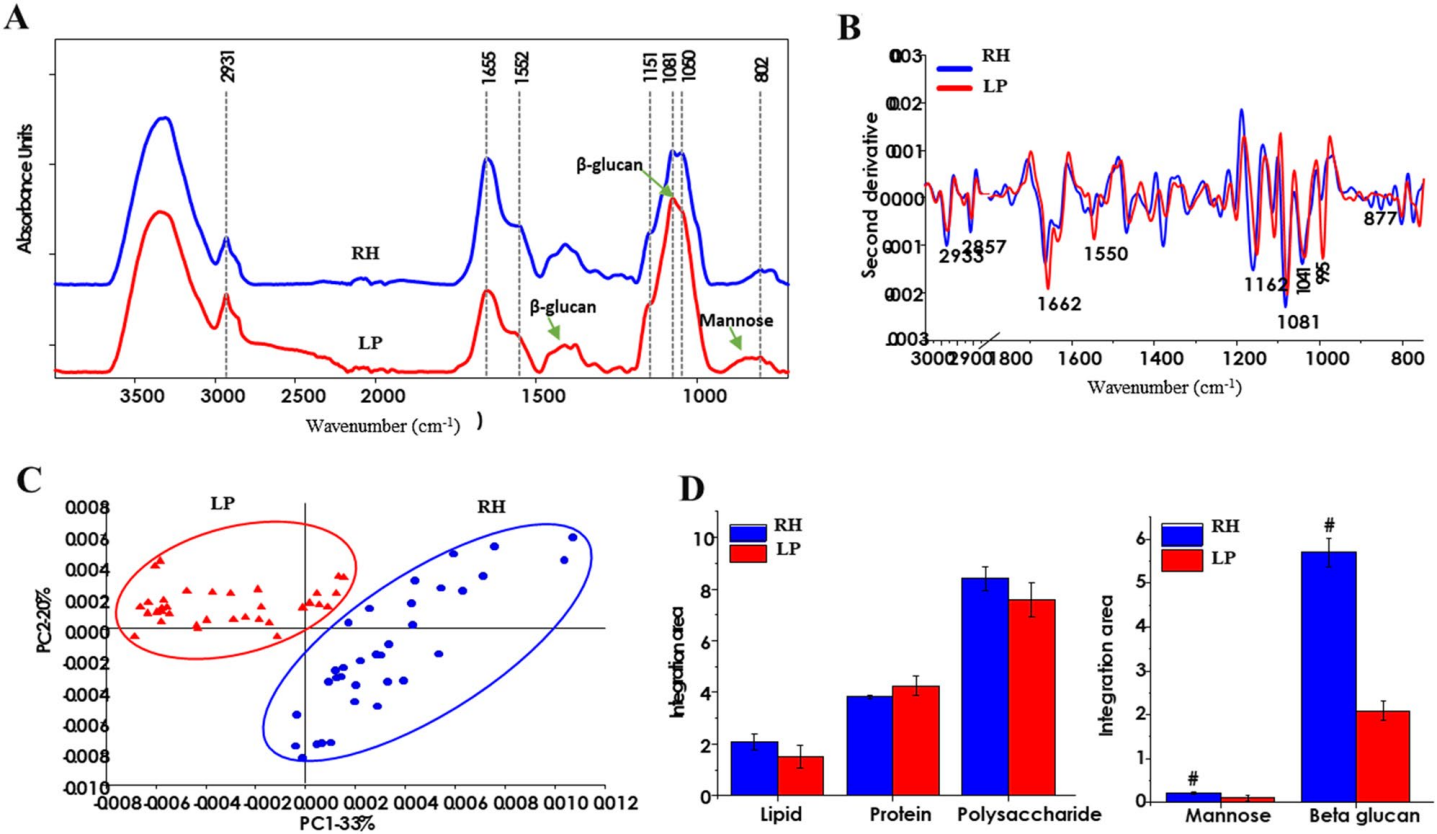
FTIR spectra (**A)**, second derivatives (**B**), principal component analysis of the FTIR-spectra (**C)**, and ratio of lipid/fat, protein and polysaccharide based on integration areas (**D)** absorbance peaks for mushroom powder (LP) and resistant hydrolysate (RH).

The effect of structural changes on carbohydrate, protein and lipid proportions was evaluated by comparing SR-FTIR spectrums of LP and resistant hydrolysates (RH), recorded in the range of 4000 to 400 cm^-1^ (Fig. 2A). It was observed that both LP and RH showed a stretching characteristic peak at 3420 cm^−1^ of hydroxyl group and the weak peak at 2935 cm^-1^ of the stretching vibration of C–H of the methylene group of aliphatic compounds from lipid and phospholipid. The peaks in 1700–1600 were due C=O, C–N stretching of amide I and those in 1600–1500 region were N–H and C–N stretching of amide II, which indicate the presence of proteins^25^.The bands within 1425 and 810 cm^-1^ were assigned to carbohydrates, and the bands at 1425 cm^−1^ and 1078 cm^−1^ strongly reveal high content of polysaccharide ^26^. Whereas, the band at 1078 cm^−1^ exhibits C–O stretching of β-glucans^26^, and 870 cm^−1^ indicates the characteristic absorption peak of mannose ^27^. The results confirmed that carbohydrates and protein are the dominant macronutrients in *L. squarrosulus*.

Moreover, principal component analysis (PCA) (Fig. 2C) showed two-dimensional plots by two PCA, PC1 and PC2 demonstrates overall variation between LP and RH. The distinctive clusters of LP and RH on the PCA plot indicate their variation due to compositional changes caused by the digestion. Again, second derivative loadings were used to visualize overlapping peaks in the original absorbance spectra. As it can be seen (Fig. 2B), the peaks at the fat/lipid, protein and polysaccharide fingerprint regions in LP and RH differ in terms of absorption wavelength and intensity. Indicating structural transformation of the major components during GI digestion. As a result, the ratio of polysaccharide, protein and lipid in LP and RH based on integration area of the absorbance peaks (Fig. 2D) was different. The results clarify that polysaccharide, particularly the proportions of β-glucan and mannose, and lipid significantly increased, while protein fraction reduced significantly. According to previous work, β-glucans enhance protection against infection by pathogenic bacteria and viruses^28^. Also, mannose-rich exopolysaccharide (EPS) from *Tremella mesenterica* was reported to induce the immune system through macrophage receptors^29^. Thus, simulated GI conditions affected the components of *L. squarrosulus* powder (LP) along with an increase of potential immunomodulatory agents; β-glucan and mannose. We posit a potential stimulation of intestinal immunity during digestion; however, further study is required to affirm this claim.

#### Digestion of carbohydrate and protein

Structural degradation (Fig. 1) and separation of aggregates (Fig. 2) released reducing sugars (R_S_) and lower molecular weight proteins. Before (0 h) digestion, the reducing sugars (R_S_) content was 0.19 ± 0.03 mg/mL, while after digestion in the gastric juice (stomach condition), R_S_ was 0.75 ± 0.01 mg/mL, showing 23.44% hydrolysis and 76.56% resistant of carbohydrate (Fig. 1). Subsequently, after small intestine digestion, R_S_ was 0.79 ± 0.01 mg/ml, given 25.17% hydrolysis and 74.83% resistant of carbohydrate (Fig. 1A). There was a significant increase (*P* < 0.05) in R_S_ in the gastric juice, but slight increase (*P* > 0.05) in R_S_ in the small intestine fluids. This means that glycoside bonds were destroyed in the gastric juice, possibly due to the low pH. However, pancreatic α- amylase had a limited effect on carbohydrate in the mushroom powder after passing through the gastric juice. In addition, the protein content (%) prior (0 h) to digestion was 30.84 ± 0.81, whereas after digestion, protein (12.24 ± 0.70) substantial (*P* < 0.05) reduced in gastric juice, and a slight reduction but no significant (*P* > 0.05) of protein (11.91 ± 0.68) recorded in the small intestine fluid. This indicates 58.59% hydrolysis and 41.41% resistant in the gastric juice, and 59.79% hydrolysis 40.21% resistant in the intestine fluids (Fig. 1B). There was a high protein digestion in the gastric juice, which can be attributed to the low pH and activity of the pepsin. Human digestion is a strong break-down process which transforms complex molecules present in foodstuffs into simpler structures easier to be assimilated by intestinal enterocytes^30^. However, structural carbohydrate such as polysaccharides and polysaccharide-protein complexes in mushroom like LP (Table 1) can resist the effect of GI conditions and reach the large intestine safely.

### In vitro colonic fermentation

#### Probiotic bacteria growth enhancement

**Figure 3:**
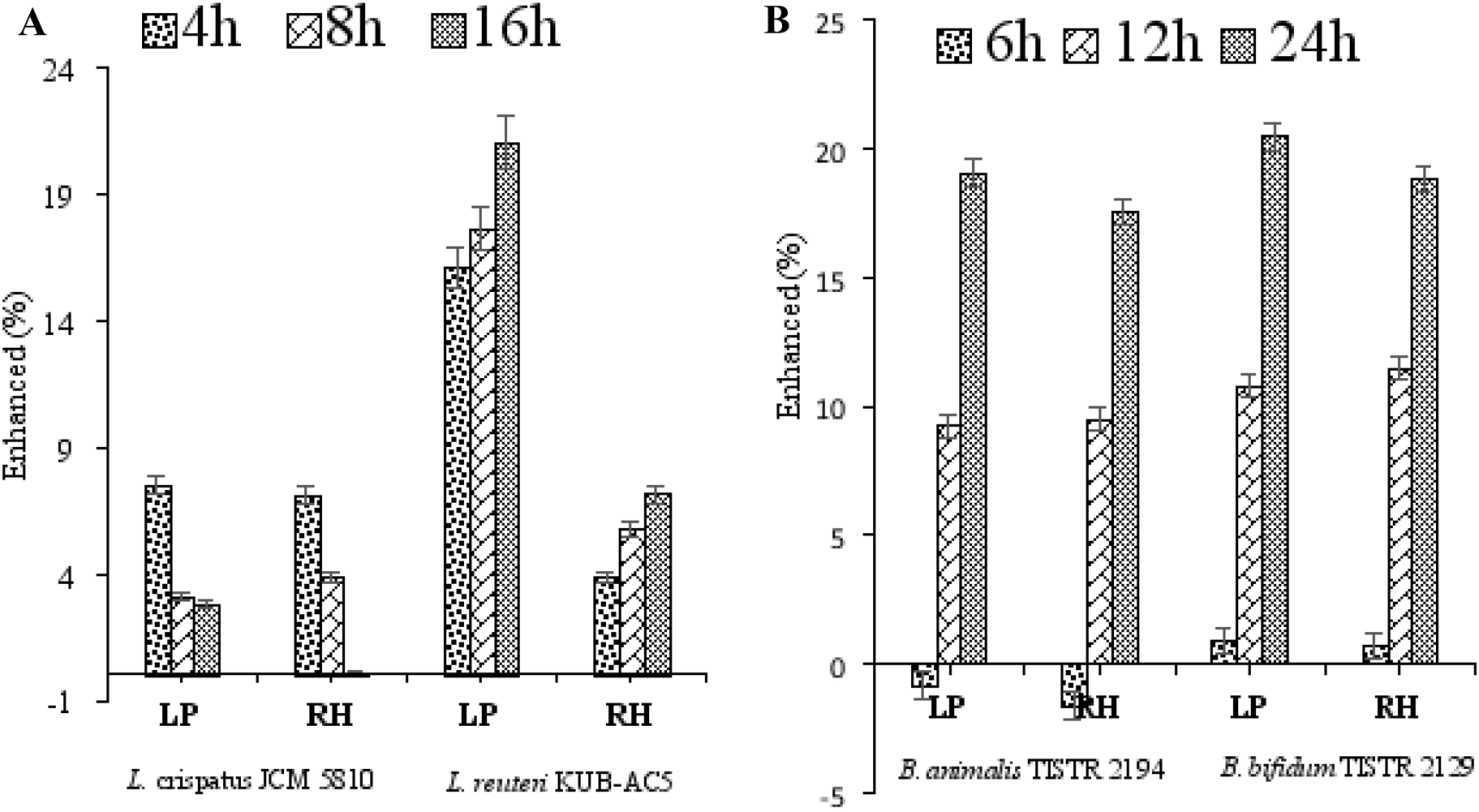
The percentage enhancement of probiotic strains by mushroom powder (LP) and resistant hydrolysate (RH) at different time points during fermentation. The values calculated on the basis of enhanced activity (taken as 100%) for the probiotics showed statistical significance (*p* ≤ 0.05) in Student’s t test.

The ability of LP and RH to enhance the growth of *Lactobacilli* (Fig. 3A) and *Bifidobacterium* (Fig. 3B) was determined and compared by measuring colony forming units (CFUs/mL) at 0, 4, 8 and 16 h, and 0, 6, 12 and 24 h of incubation, respectively. All probiotic strains were significantly enhanced (%) (*p* ≤ 0.05), and showed different growth characteristics in dependence on sample and strain specificity. As presented in Fig. 3A, maximum growth enhancement of *L. crispatus* JCM 5810 was reached at 4 h, similarly toward LP and RH but greatly reduced thereafter, especially with RH. Whereas, *L. reuteri* KUB-AC5 was continually enhanced by LP and RH from 4 to 18 h in a comparable trend, but better with LP. Moreover, *B. bifidum* TISTR 2129 showed a negative growth enhancement, while *B. animalis* TISTR 2194 was positively promoted after 4 h by LP and RH as shown in Fig. 3B. However, both bacteria were substantially enhanced after 8 and 16 h in a similar manner but slightly better with LP. It can be inferred that growth enhancement of *L. reuteri* KUB-AC5, *B. bifidum* TISTR 2129 and *B. animalis* TISTR 2194 were more sensitive toward LP than RH. Also, RH promoted *Bifidobacterium* better than *Lactobacilli*. Thus, indigestible carbohydrates and proteins are important energy sources for the human gut microbiota. Hence, this information may serve as a basis to evaluate how RH can stimulate the human gut microbiota.

### Human gut microbiota fermentation of resistant hydrolysate (RH)

#### Effect of RH on richness and diversity of human microbiota

**Figure 4:**
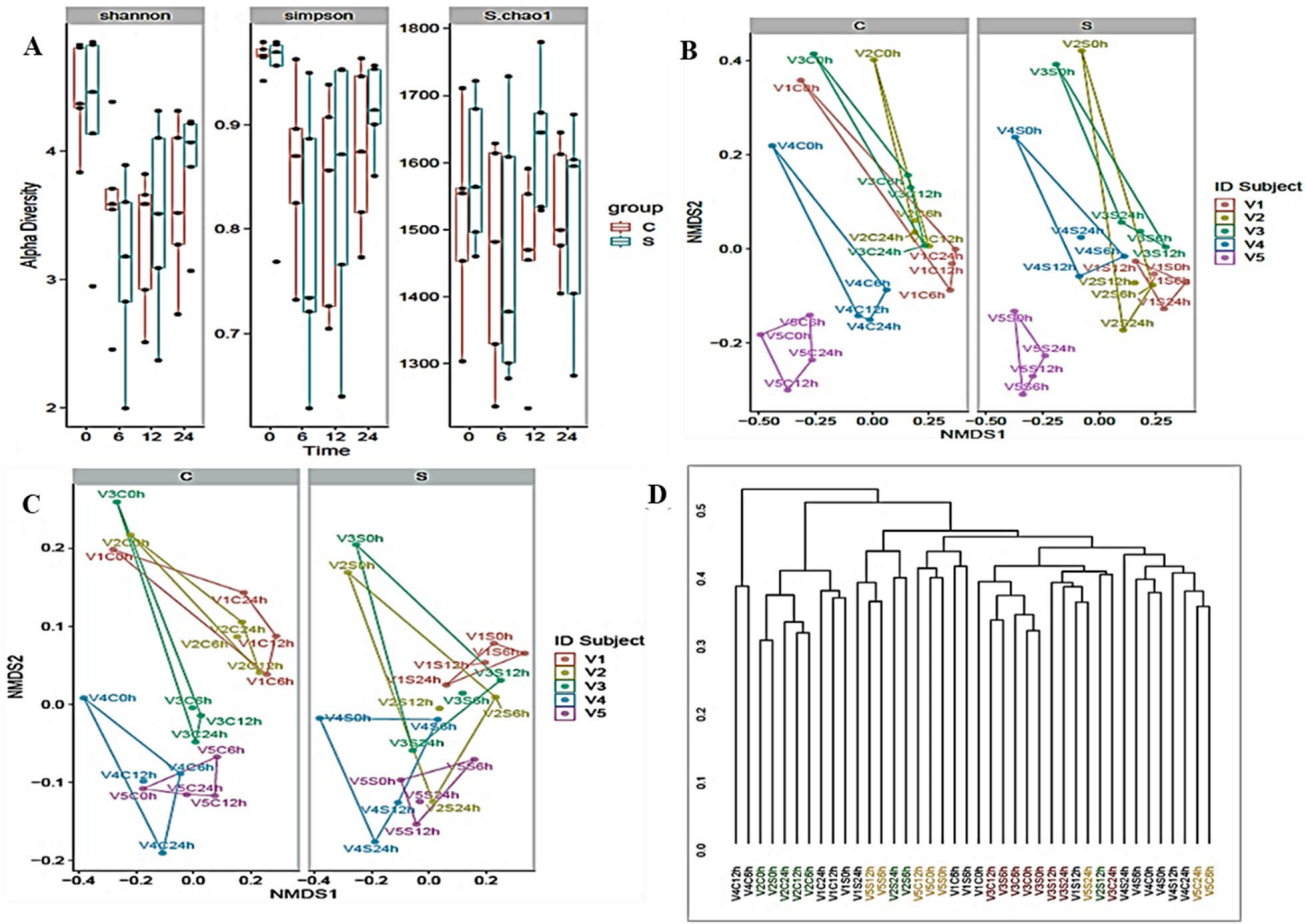
Composition of gut microbiota. (**A**) Alpha diversity indexes of samples in control groups and samples (S) or resistant hydrolysate (RH) treated groups, (**B**) Nonmetric multidimensional scaling (NMDS) analysis of microbiota based on Jaccard distance, (**C**) Nonmetric multidimensional scaling analysis of microbiota based on wunifrac distance, and (**D**) Multivariate analysis of variance from matrix scores based on Bray–Curtis method. Significant differences were computed among different time points within control and RH sample groups of volunteers (V1, V2, V3, V4, V5).

The study investigated the effects of resistant hydrolysate (RH); sample (S) obtained after digesting *L. squarrosulus* powder in simulated human upper gut (stomach and small intestine) on gut microbiota by high throughput sequencing analysis. S. chao 1 revealed the richness of the different microbiota. In Fig. 4A, the richness of the microbiota before fermentation differs significantly among individual volunteers, V2 appeared the richest. It was followed by V1 and V3, which were similar (*p* > 0.05) and lastly, V4 and V5, which seems to be comparable. Also, the richness of bacterial between the control (C) and RH treated (S) groups for each individual microbiota during 24 h fermentation varied (*p* < 0.05). RH advanced bacterial richness in V1, V2 and V4 at 12 h, and also, in V5 at 12 h and 24 h. Whereas, in V3, RH slightly improved bacterial richness at 24 h. The supplement of RH might support the growth of the least populated bacterial group, which could improve bacterial richness of individual gut microbiota. The Shannon and Simpson indexes signified the diversity of the various microbiota. As shown in Fig. 4A, at 0 h, bacterial diversity between the control and treatment groups were comparable for the individuals. V2 and V3 had a comparable, with the highest diversity. Following this, V4 and V5 showed a similar, with the second highest diversity, while V1 exhibited the least diversity. During fermentation, bacterial diversity dropped in V1, V2 and V3 from 6 to 24 h but RH groups exhibited a higher diversity. However, RH maintained bacterial diversity in V4 and V5, especially at 12 h and 24 h. Even though, a healthy human gut microbiota has not yet defined at any profound taxonomic resolution. However, bacterial diversity and richness are generally considered indicators of healthy (eubiosis) and bad (dysbiosis) microbiota^31^. Among individuals, V2 exhibited the most rich and diverse bacterial composition and afterwards, V3 displayed the most bacterial richness and diversity. V1 showed more bacterial richness but low diversity when compared to V4 and V5. According to previous report, a healthy human colon microbiota yields a relatively high rich and diverse abundance of microbes^31^. In this study, V2 and V3 may have better or more resilience gut microbiota than V1, V4 and V5. Interestingly, RH improved bacterial richness in all the individuals but reduced bacterial diversity in V1, V2 and V3. Indicating that RH may have a significantly impact on both weak and resilient gut microbiota.

Also, the microbiota structure and composition between the control and RH groups were analyzed by NMDS-Jaccard distance and NMDS- wunifrac distance. As shown in Fig. 4B, C, volunteers between groups are clearly separated, and three volunteers; V1, V2, and V3, while two volunteers; V4 and V5 are closer to each other. A similar trend was observed in the control and RH group. However, RH group from 6 to 24 h were separated. Suggesting that V1, V2, and V3 shared more similar microbiota, and V4 and V5 also shared a comparable gut microbiota but RH influence on their similarities depend on time. Multivariate analysis of variance based on Bray–Curtis method was also used to evaluate the similarity between control and RH groups of volunteers, and the results (Fig. 4D) were consistence with the results of NMDS.

#### Effect of resistant hydrolysate on gut bacteria composition

**Figure 5:**
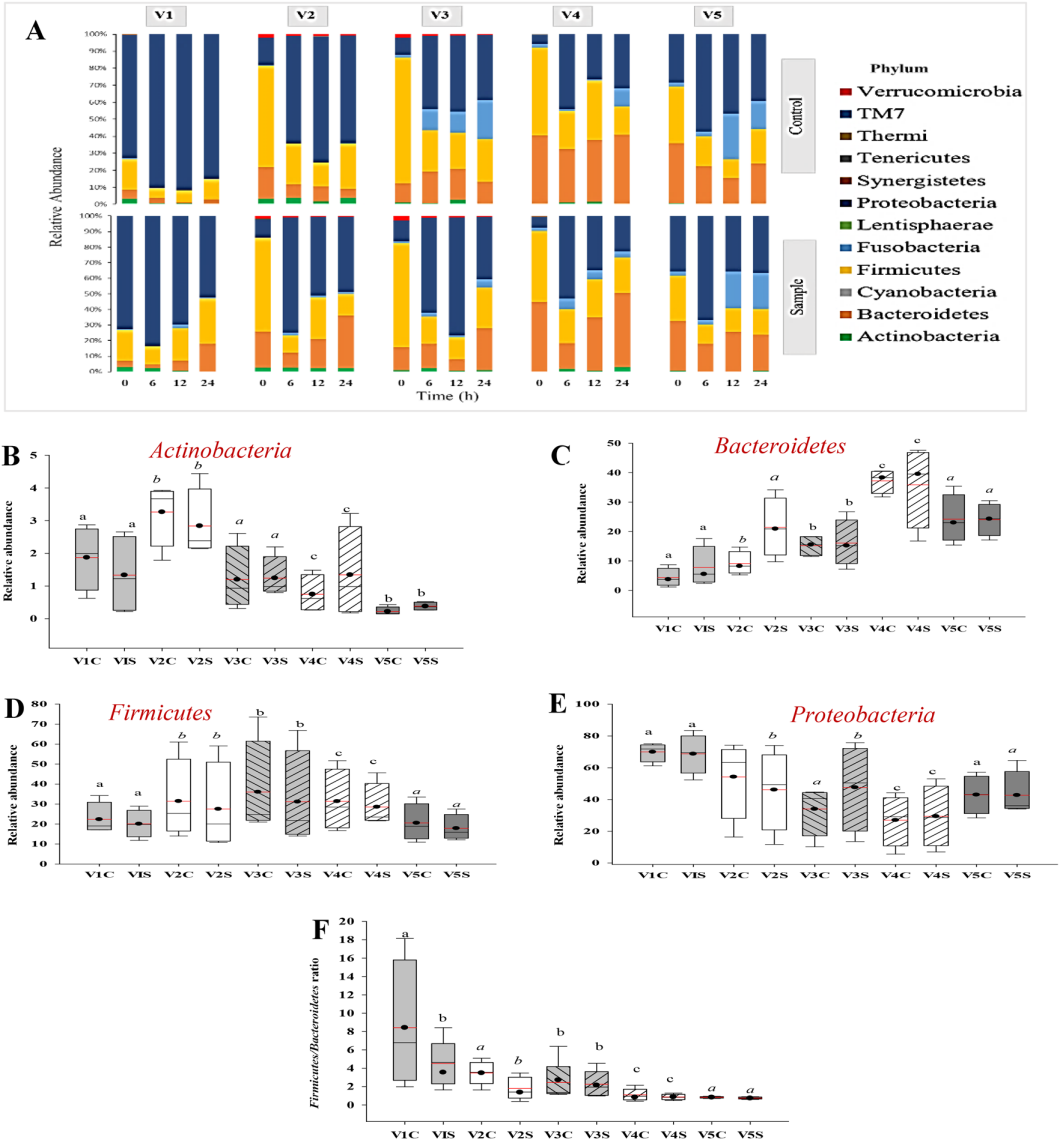
Effect of resistant hydrolysate (RH) on relative composition of gut microbiota at the phylum level. (**A**) Bacterial taxonomic profiling in the phylum level of five human gut microbiota during 24 h fermentation of resistant hydrolysate (RH). (**B**) Relative abundance of *Actinobacteria* (*p* = 0.00), (C) *Bacteroidetes* (*p* = 0.001), (**D**) *Firmicutes* (*p* = 0.00), (**E**) *Proteobacteria* (*p* = 0.00) and (**F**) the ratio of *Firmicutes*/ *Bacteroidetes*. Different lowercase shows significant difference (*P* < 0.05) of bacterial population in different groups.

The structures of the gut microbiota of five volunteers (V1, V2, V3, V4 and V5) were investigated to compare the effects of resistant hydrolysate (RH) on the intestinal microbes. Figure 5 presented the gut microbial composition at phylum level before and after fermentation with (as control; C) or without RH (S). At 0 h, more than 97% of the total sequences detected in all samples belonged to the five most abundant bacterial phyla, *Proteobacteria* (*p* = 0.00)*, Bacteroidetes* (*p* = 0.001)*, Firmicutes* (*p* = 0.00)*, Actinobacteria* (*p* = 0.00) and *Fusobacteria* (*p* = 0.208) with significant differences among the five volunteers (Fig. 5A). V1 contained more of *Proteobacteria* (70.56%) and *Firmicutes* (18.04%). V2 was abundant in *Firmicutes* (60.08%) and *Bacteroidetes* (18.84%). V3 composed of mainly *Firmicutes* (70.25%) and *Bacteroidetes* (13.13%). V4 consisted of *Firmicutes* (48.68%) and *Bacteroidetes* (42.32%). V5 was made-up of comparable abundant of *Firmicutes* (32.95%), *Bacteroidetes* (30.28%) and *Proteobacteria* (31.22%). *Actinobacteria* was significantly different (*p* = 0.000), which was abundant in V2 (3.98%) and V1 (2.51%) but significantly lower and similar among V3, V4 and V5. Whereas, *Fusobacteria* abundant detected was not significantly different (*p* = 0.208) among the volunteers. Additionally, *Firmicutes*/*Bacteroidetes* ratio for V1, V2, V3, V4 and V5 was 4.7, 3.2, 5.3, 1.1 and 0.9, respectively, which were significantly different (*p* = 0.00). This means that V1, V2 and V3 may have more risk of obesity than V4 and V5.

In comparison with the control during fermentation, RH addition significantly increased the relative abundance of *Bacteroidetes* in the gut microbiota of V1 and V2 (*p* < 0.05) (Fig. 5C), whiles *Firmicutes* were comparable (*p* > 0.05) but showed a trend of decreasing in RH treated groups (Fig. 5D). As a result, RH greatly (*p* < 0.05) reduced the ratio of *Firmicutes/Bacteroidetes* (F/B) (Fig. 5F). The F/B ratio after 24 h fermentation in the control of V1, V2, V3, V4 and V5 was 8.76, 5.09, 1.98, 0.41 and 0.84, while in RH groups was 1.64, 0.39, 0.98, 0.48 and 0.71, accordingly. Notably, when compared to the control during fermentation, RH significantly reduced F/B in V1, V2, V3, V4 and V5 by 5.3, 13.1, 2.0, 0.9, 1.2 times, respectively. Again, when compared to before fermentation (0 h), RH significantly decreased F/B in V1, V2, V3, V4 and V5 by 2.9, 8.2, 5.4, 2.3 and 1.3 times, orderly. In addition, the relative abundance of *Actinobacteria* between RH and control blank groups were not significantly different (*p* > 0.05) in gut microbiota of all volunteers (Fig. 5B). Meanwhile, the addition of RH significantly decreased the abundance of *Proteobacteria* in V1, V2 and V4 (*p* < 0.05) (Fig. 5E), but did not significantly change (*p* > 0.05) *Proteobacteria* abundance in V3 and V5 when compared to the control after 24 h. according to previous reports, the *Firmicutes*/*Bacteroidetes* ratio is linked with obesity, and was positively associated with a reduced energy harvest. However, a reduction of *Firmicutes*/*Bacteroidetes* signified intestinal health and weight loss^32^. Also, in another report, increasing the abundance of species of *Proteobacteria* was recognized as a potential microbiological characteristic of diseases, such as inflammatory bowel disease (IBD), metabolic disorders and lung diseases^33^. Indicating that RH improved the gut microbiota of all the volunteers by decreasing F/B ratio and *Proteobacteria* abundance. Hence, *L. squarrosulus* powder (LP) has a potential to stimulate the gut microbiota of human and may reduce the risk of obesity and control gut related diseases.

#### Gut microbiota composition analysis at genus level

**Figure 6:**
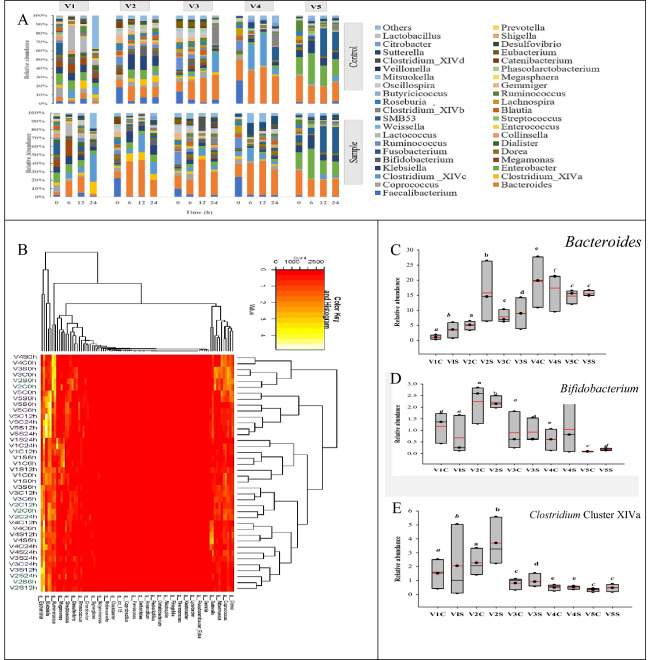
Impacts of resistant hydrolysates (RH) on the compositions of gut microbiota of five volunteers at the genus level. (**A**) Relative abundance of bacteria at the genus level. (**B**) Heatmap analysis at the genus level. (**C**) Relative abundance of *Bacteroides*. (**D**) Relative abundance of *Bifidobacterium*. (**E**) Relative abundance of *Clostridium*. Different lowercase shows significant difference (*P* < 0.05) of bacterial population between RH treated and control group for each volunteer.

A total of 92 kinds of genera (Fig. 6A) were identified at the genus level, comprising *Bacteroide, Bifidobacterium*, *Clostridium*_XlVa, *Megamonas, Dialister, Prevotella, Faecalibacterium, Dialister, Ruminococcus* and others. For better clarity of comparing difference between groups, the genera with relative abundance above 0.1% were analyzed. Ultimately, 41 genera in the gut microbiota of volunteers were designated for the subsequent analysis (Fig. 6B). As it can be noted, RH fermentation significantly reduced (*P* < 0.05) the relative abundance of *Phascolarctobacterium* and *Prevotella* in the gut microbiota of all volunteers (V1, V2, V3, V4 and V5) compared to the control. *Phascolarctobacterium* reduction occurred at all time points in V1, V2, V3 and V5, but at 12 h and 24 h in V4. Whereas, *Prevotella* decreased after 24 h in V1, V3 and V5 but at 12 h and 24 h in V4. Also, the relative abundance of *Coprococcus* and *Catenibacterium* significantly decreased in RH group of V1, V2, V3 and V5. While, *Gemmiger* significantly decreased in V1, V2, V3 and V4. Again, the relative abundance of *Faecalibacterium* and *Megamonas* significantly declined in RH groups. *Faecalibacterium* decreased in V2, V3 and V5, while *Megamonas* reduced in V2, V3 and V4.

Meanwhile, 27 genera were significant (*p* > 0.05) increased in RH treatment groups combined, compared to the control groups. The relative abundance of *Bacteroides*, *Bifidobacterium*, *Clostridium* cluster XIVa and IV, and *Sutterella* were significantly (*P* > 0.05) improved in the gut microbiota of all volunteers but at different time points. *Bacteroides* increase occurred at 6 h and 12 h of fermentation in V1, 6 h and 24 h in V2, 12 h in V3, and 6 h in V4 and V5. *Bifidobacterium* was improved only at 6 h in V2 microbiota, and 24 h in the gut microbiota of V1, V3, V4 and V5. *Clostridium* cluster XIVa and IV were enriched at 6 h and 24 h fermentation in V2 and V5, 12 h and 24 h in V1, V3 and V4. High abundance of *Sutterella* was observed at all time points in V1, V3, V4, but at 12 h in V2 and at 12 h and 24 h in V5. Also, the relative abundance of *Dialister* were significantly enriched (*p* < 0.05) in four volunteers at different time points. *Dialister* was significantly enriched at 12 h fermentation of RH in V1 and at 24 h in V2, V3 and V5. Again, RH significantly increased the abundance of *Ruminococcus*, *Desulfovibrio*, *Collinsella* and *Citrobacter* in three of the volunteers. While *Ruminococcus* and *Lachnospira* were improved in V1, V4 and V5, *Citrobacter* in V1, V2 and V3, *Desulfovibrio* in V1, V2 and V5, *Lactococcus* after 24 h in V2 and V4. Moreover, RH significantly enriched abundance of *Streptococcus* in V3 and V4, *Collinsella* in V4 and V5, *Oscillospira* in V1 and V4. *Fucobacterium* 24 h fermentation of RH in V2 and 12 h in V4. Furthermore, RH significantly improved *Megamonas* and *Mitsuokella* in V1 and V5, *Enterococcus* in V2, *Roseburia*, *Blautia*, *Lactobacillus*, *Catenibacterium* and *Eubacterium* in V4, and *Veillonella* in V5. In particular, the relative abundance of *Bacteroides* (Fig. 6C), *Bifidobacterium* (Fig. 6D), *Clostridium* cluster XIVa and IV (Fig. 6E) were significantly improved in the gut microbiota of all volunteers.

*Bacteroides* are main components of the phylum *Bacteroidetes* in the gut microbiota. They have carbohydrate-active enzymes that catalyzed the acquisition and metabolism of several glycosides. RH supplementation caused *Bacteroides* to become the dominant genus in V1, V2, V3 and V4 after 24 h of fermentation. *Bacteroides* have potential health benefits in hosts by lowering intestinal inflammatory responses and enhancing intestinal stability^34^. Besides, current research has revealed that *Bacteroides,* which decrease the expression of angiotensin-converting enzyme 2 (ACE2) in murine gut, correlated negatively with SARS-CoV-2 load in fecal samples taken from patients^35^.

*Bifidobacterium* as an acid-producing bacteria, is a commonly used probiotic that contribute importantly to host health^36^. *Bifidobacterium* has a potential to inhibit pathogenic microorganisms in infants^37^, protect the host from inflammatory diseases^38^, and improve disease symptoms in mice of the T-cell transfer colitis mode^39^. Also, in recent report, *Bifidobacterium* has known immunomodulatory bacterial remained low in COVID-19 patients, even after disease resolution^40^. RH enriching *Bifidobacterium* in the gut microbiota of all volunteers may release metabolites, activate immunomodulatory pathway, and finally reduce the risk of diseases.

It was found that *Bifidobacterium* initiate oligofructose degradation, with a relation production of acetate, while some of the *Clostridium* cluster XIVa and IV bacteria in the colon are capable of degrading simultaneously oligofructose and associated consumption of acetate, resulting in the production of butyrate. Also, *Clostridium cluster XIVa* are capable of converting lactate to butyrate. In this work, *Clostridium* cluster XIVa and IV were enhanced in the gut microbiota of all volunteers when RH was added. This signified cross-feeding, which indicates direct stimulation of butyrate-producing colon bacteria in these hosts by RH.

*Dialister*, together with *Coprococcus* were reported depleted in people with important indicators of depression^41^. We observed RH improved the abundance of *Dialister* in the microbiota of four volunteers, V1, V2, V3 and V5. Suggesting that RH may potentially help to condition human gut microbiota to fight depression. However, further research is needed to confirm this claim.

In addition, RH promoted *Ruminococcus*, *Desulfovibrio*, *Collinsella, Citrobacter, Streptococcus* and *Mitsuokella* in at 3 volunteers, while *Lactococcus*, *Oscillospira*, *Collinsella, Fucobacterium*, *Eubacterium* were improved in at least 2 volunteers. Other genara, *Megamonas, Roseburia*, *Blautia*, *Lactobacillus* and *Catenibacterium* were enhanced in at least 1 volunteer. Some of these groups such as *Roseburia*, *Lactobacillus*, *Megamonas*^42^*, Oscillospira*^43^ and *Lactococcus*^44^ have been reported to contribute to host health. *Streptococcus*^45^ and *Eubacterium*^46^ were indicated to confer beneficial effects on obesity and inflammation. The results showed that RH produced positive changes in the gut microbiota, but these depend on the initial composition of a volunteer’s gut microbiota. Notably, RH enhanced the relative abundance of known immunoregulatory bacteria, such as *Bacteroides*, *Bifidobacterium* and *Clostridium* cluster XIVa and IV. This signifies a potential application to stimulate an altered gut microbiota to reduce gut related diseases.

#### Microbial profile of the gut microbiota

**Figure 7:**
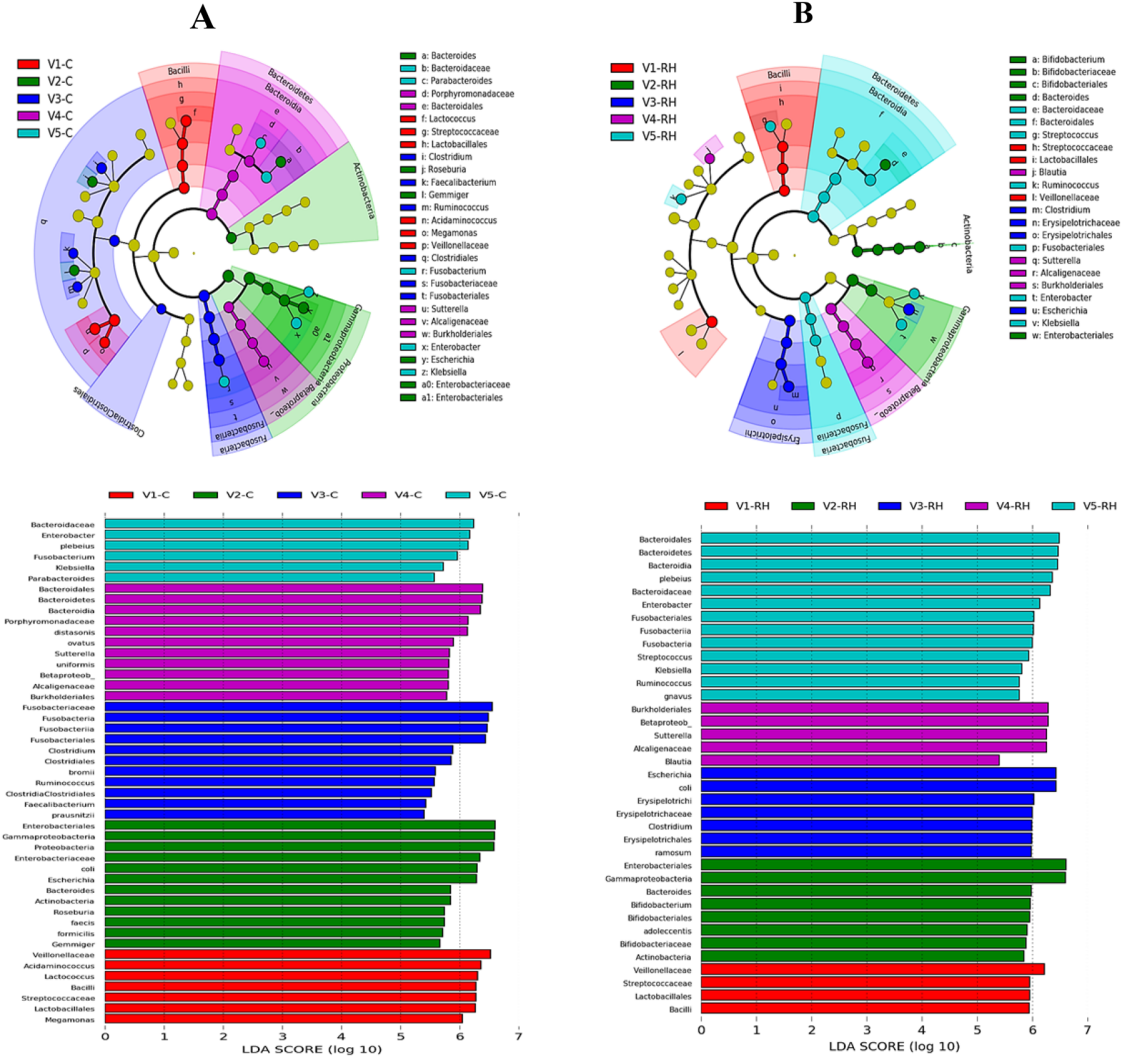
Effects of resistant hydrolysate (RH) on dominant microorganisms based on linear discriminant analysis effect size (LEfSe). Cladogram and Distribution histogram based on LDA; control (C); A, RH; B.

Linear discriminant analysis (LDA) effect size (LEfSe) was performed to illustrate the differences in the microbial profiles of the gut microbiota of volunteers between control (A) and RH treated group (B). All the volunteer microbiota in the control and RH group were mainly consisted of *Bacteroidetes*, *Proteobacteria*, *Firmicutes* and *Actinobacteria* (Fig. 7). Based on the results, there was a major difference between the control and RH group. Also, the effect RH on relative abundance of bacteria was volunteer specificity. In comparison with the control, at phylum level, RH increased the relative abundance of *Bacteriodetes* and *Fusobacteria* in the microbiota of V5. At order level, *Bifidobacteriales* were improved by RH in V2, while relative abundance of *Erysipelotrichales* was increased by RH in V3. At family level, RH promoted the relative abundance of *Bifidobacteriaceae* in V2, whereas in V3, the relative abundance of *Erysipelotrichales* was elevated by RH. At genus level, RH enriched the relative abundance of *Bifidobacterium* in V2, *Escharichia* in V3, *Blautia* in V4, and *Streptococcus* and *Ruminococcus* in V5. Notably, RH seems to enriched more bacteria groups in V5 than the other volunteers. In all, the results were in accordance with the gut microbiota composition analysis.

#### The effect of RH on metabolites production

**Figure 8:**
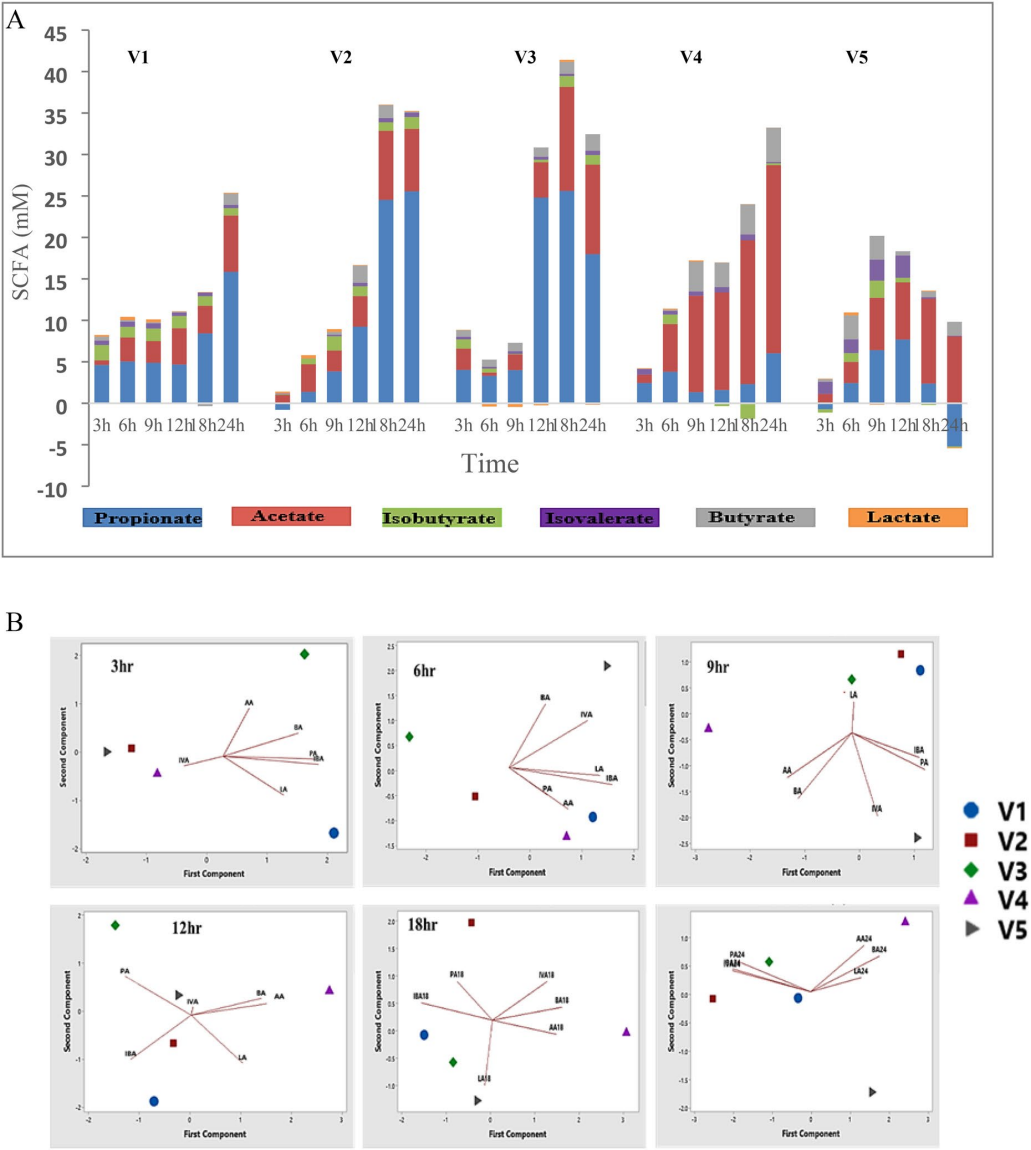
(**A**) Relative change of short-chain fatty acid (SCFA) and branched-chain fatty acid (BCFA) concentrations (mmol (mM)/mL) during 24 h fermentation of mushroom hydrolysate compared to the control among five individuals. (**B**) Biplot relating SCFA and BCFA to the fecal sample from five individual. Among individuals, *P-value* of SCFA: Lactic acid; LA (*0.062*), acetic acid; AA (*0.001*), propionic acid; PA (*0.042*), butyric acid; BA (*0.007*), isovaleric acid; IVA (*0.023*) and iso-butyric acid; IBA (*0.021*).

Lactic acid, short-chain fatty acids (SCFA) and branch-chain fatty acids (BCFA) concentrations during 24 h fermentation of resistant hydrolysate (RH) of fecal inocula from five healthy volunteers were evaluated. The RH was fermentable in the gut microbiota of all volunteers. Propionic, acetic, lactic, butyric, isovaleric and iso-butyric acids were detected in RH and control treatments (Fig. 8A). Interestingly, the concentrations of lactic acids, SCFAs and BCFAs were significantly (*p* < 0.05) increased by the addition of RH at different time points compared to the control. Hence, RH influenced the production and richness of SCFAs and BCFAs metabolites. However, the increased in richness of lactic acids, SCFAs and BCFAs were dependent on volunteer and time (3 h, 6 h, 9 h, 12 h, 18 h and 24 h). After fermentation, lactic acid richness did not vary significantly (*p* > 0.05) among volunteers at various time points. Lactic acid concentration (mM/mL) fluctuated between 0.059–0.387, −0.295–0.267, −0.448–0.228, 0.014–0.145 and −0.173–0.344 for V1, V2, V3, V4 and V5, respectively. The increased lactic acid during RH fermentation was stable in V1 and V4 than the others. Meanwhile, the richness of SCFAs including acetic, propionic and butyric acids during RH fermentation differed significantly among volunteers and at various time points for each volunteer. The concentration (mM/mL) of SCFAs ranged between 5.6–24.01, 0.25–33.17, 4.56–39.60, 3.45–32.84 and 0.71–15.58 for V1, V2, V3, V4 and V5, accordingly. Notably, propionic was the most increased SCFA in V1, V2 and V3, while acetic acid was the most enhanced in V4 and V5. The proteolytic fermentation produces less SCFAs, but also branched-chain fatty acids BCFAs, including isovaleric and iso-butyric acids^47^. In this study, the richness of BCFAs, consisting isovaleric and iso-butyric acids did not differ significantly among volunteers during fermentation of RH. The BCFA concentration (Mm/mL) ranged between 1.28–2.38, 0.16–1.99, 0.38–1.69, −1.15–1.59, −0.02–4.61 for V1, V2, V3, V4 and V5, respectively.

Moreover, in order to better visualize the influence of RH on production of SCFAs in gut microbiota of volunteers, PCA-Biplot analysis was employed. The biplot for first and second components showed the impact of RH on individual acids in the excreta of five volunteers at 3 h, 6 h, 9 h, 12 h, 18 h and 24 h fermentation (Fig. 8B). The visualization with use the plot showed a distinct correlation of individual acids with the gut microbiota of the volunteer’s dependence on time.

Several studies exhibited biological activities of SCFAs produced by gut bacteria. Briefly, SCFAs could promoted gastrointestinal peristalsis, protect intestinal barrier, reduce the inflammation and colon cancer level ^48^. Indigestible carbohydrates could be fermented to produce organic acids, such as acetic, propionic, butyric and lactic acids by most intestinal microorganisms^49^. Also, proteins, such as branched chain amino acids and peptides, as fermentation substrate, could further be converted to BCFAs by gut bacteria^50^. After GI digestion of LP, RH consisted of about 70% carbohydrate and 40% protein (Fig. 1). Interaction of RH with the gut bacteria of five volunteers resulted in an improved richness of SCFAs and BCFAs compared to the control (Fig. 8A). Obviously, lactic acid and BCFAs richness was not significantly different among volunteers. Meanwhile, richness of SCFAs such as propionic, acetic and butyric acids were significantly different among the five volunteers. Hence, these acids set the difference on which the gut microbiota of volunteers used RH to produce metabolites. V1, V2 and V3 seem to used RH and convert it to more of propionic but less butyric acids compared to V4 and V5 that fermented RH to released more of acetic and butyric acids.

By far, different functions of lactic acid, BCFAs and SCFAs have been emphasized, thus the potential functions of RH can be extrapolated. Propionic acid plays keen role in weight loss, anti-inflammation and lowering cholesterol, and the protection against diet-induced obesity and regulation of gut hormones^51^. Butyrate acid, the most greatly highlighted SCFA, is the basic fuel source for colon cells and contributes to the protection against colorectal cancer, inhibition of inflammation, increasing thermogenesis and resistance to obesity^52^. Acetic acid function in lowering the accumulation of abdominal fat and protecting against the accumulation of lipids in the liver^53^. The isovaleric and iso-butyric acids, with very low content, might be formed from pathways of indigestible protein fermentation, may play important roles in the gut environment^54^. Lactate is a precursors of propionate and can be conversed to propionate through acrylate pathway^55^, and could the reason for it fluctuation during fermentation. It can be inferred that the protein and carbohydrate fractions in RH can be fermented in the gut microbiota all the volunteers to enrich beneficial organic acid profiles. However, the conversion of carbohydrate fraction to propionic, acetic and butyric acids depends on the individual’s microbial ecology.

#### Correlation between lactic acid, SCFA and BCFA and microbiota

**Figure 9:**
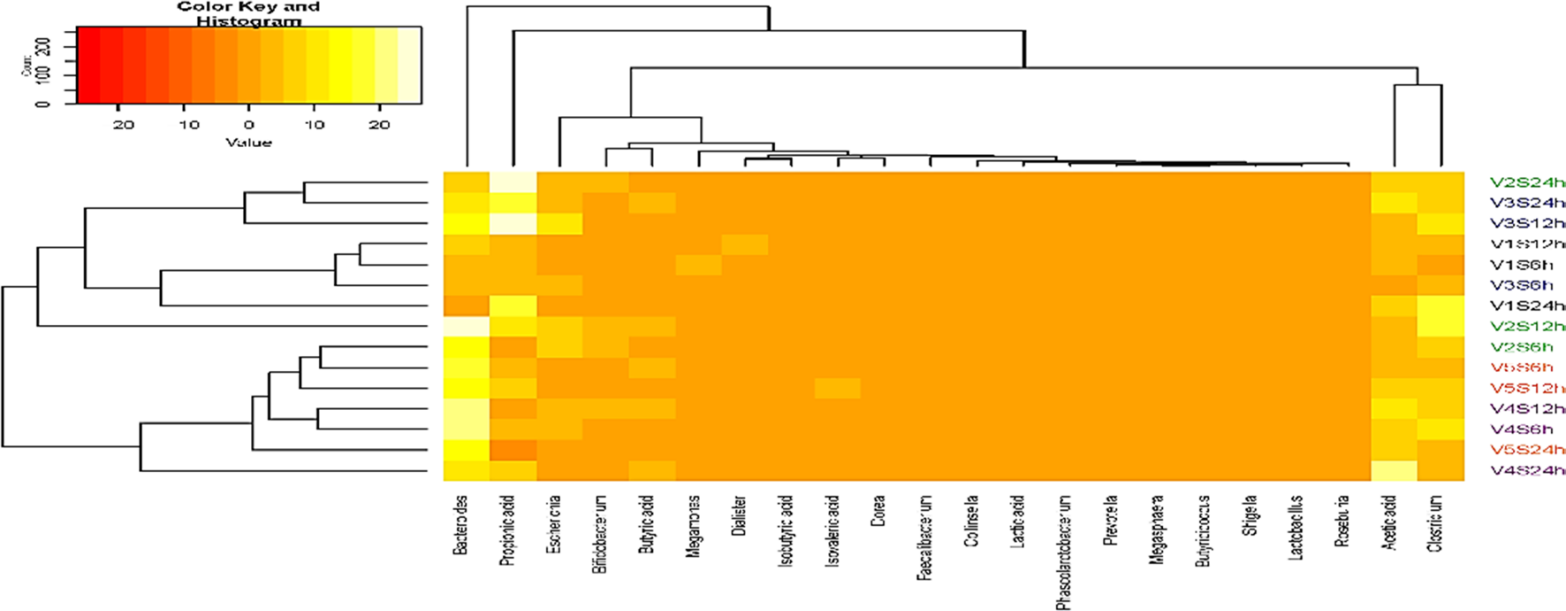
Correlation between 6 kinds of acids and microbiota at genera level (relative abundance in V1, V2, V3, V4 and V5 groups) according to time. The fill color shows the direction of a correlation (yellow for positive and red for negative). The five volunteers (V): V1 (V1S6h, V1S12h, V1S24h); V2 (V2S6h, V2S12h, V2S24h); V3 (V3S6h, V3S12h, V3S24h); V4 (V4S6h, V4S12h, V4S24h); V5 (V5S6h, V5S12h, V5S24h) treated with resistant hydrolysate (RH) of *L. squarrosulus*.

As clearly shown in Fig. 9, the relative abundance of *Megamonas*, *Dialister*, *Dorea*, *Faeclibacterium*, *Collinsella*, *Phascolarctobacterium*, *Prevotella*, *Megasphaera*, *Butyricicoccus*, *Shigella*, *Lactobaccillus* and *Roseburia*, and lactic, isovaleric and butyric acids have strong similarities among individual volunteers with respect to time. However, V1S12h and V1S6h showed distinct relative abundance of *Dialister* and *Megamonas,* orderly, while V5S12h resulted in a unique concentration of isovaleric acid. Also, the relative abundance of *Bifidobacterium* is closely related with butyric acid and showed a strong positive dissimilarity among volunteers. Whereas, *Escherichia* does not closely relate with any of the acids but showed a strong positive dissimilarity among volunteers. Meanwhile, relative abundance of *Bacteroides* and *Clostridrium* closely relate with propionic and acetic acids, respectively, with a very strong positive dissimilarities among volunteers over time. Thereinto, volunteer 1(V1) group was abundance in *Clostridium*, *Megamonas* and *Bacteroides* in V1S6h, *Clostridium*, *Dialister* and *Bacteroides* in V1S12h, and *Bacteroides* and *Clostridium* in V1S24h. Volunteer 2 (V2) group was abundance in *Clostridium, Bifidobacterium*, *Escherichia* and *Bacteroides* in V2S6h, V2S12h and V2S24h. Volunteer 3 (V3) group was abundance in *Clostridium*, *Escherichia* and *Bacteroides* in V3S6h, V3S12h and V3S24h. Volunteer 4 (V4) group was abundance in *Clostridium*, *Escherichia* and *Bacteroides* in V4S6h, *Clostridium*, *Bifidobacterium*, *Escherichia* and *Bacteroides* in V4S12h, and *Clostridium* and *Bacteroides* in V4S24h. Volunteer 5 (V5) group was abundance in *Clostridium* and *Bacteroides* in V5S6h, V5S12h and V5S24h. Notably, V1S6h and V1S12h showed a positive correlation with acetic and propionic acids, while V1S24h also showed a very strong positive relation with these acids. In V2 group, V2S6h showed a strong positive correlation with acetic and propionic acids, while V2S12h showed acetic, propionic and butyric acids. V2S24h had a strong positive correlation with acetic acid but an extremely strong correlation with propionic acid. In V3 group, V3S6h positively correlated with acetic and propionic acids, likewise V3S12h positively linked with acetic acid but with an extreme correlation with propionic acid. V3S24h showed a very strong correlation with acetic and propionic acids. In V4 group, V4S6h had a strong positive relation with acetic and propionic acids, while V4S12h showed a very strong correlation with acetic and butyric acids. V4S24h had an extreme positive correlation with acetic and with a strong positive correlation with propionic and butyric acids. Moreover, V5S6h had a strong positive correlation with acetic acid and a very strong positive correlation with butyric and propionic acids, while V5S12h showed a very strong positive correlation with acetic, isovaleric and propionic acids. Whereas, V5S24h only showed a very strong positive correlation with acetic acid. Notably, high abundance of *Clostridium* in V1, V2 and V3 from 12 to 24 h correlated strongly with propionic acid richness. Whereas, its less abundance in V4 and V5, correlated strongly with acetic acid. This means *Clostridium* might use acetic acid as precursor to produce other acids, which is in line with gut microbiota composition analysis. In general, RH enhanced the growth of beneficial bacteria along with the production of SCFAs and BCFAs known to ensure a balanced gut microbiota, but influenced by the baseline microbiota of the individuals.

## Conclusion

GI conditions altered the structure and ratio of micronutrients in LP, and 71.67% carbohydrate and 40.41% protein of RH could reach the colon safely. With fermentation by probiotic bacteria, *L. reuteri* KUB-AC5, *B. bifidum* TISTR 2129 and *B. animalis* TISTR 2194 were significantly enhanced but were better with LP. RH was fermented by the fecal microbiota of five volunteers. RH significantly decreased *Firmicutes*/*Bacteroidetes* ratio in V1, V2, V3, V4 and V5 by 2.9, 8.2, 5.4, 2.3 and 1.3 times, respectively. Also, the relative abundance of *Bacteroides*, *Bifidobacterium*, *Clostridium* cluster XIVa and IV, and *Sutterella* were enriched by RH, which was dependent on time. Moreover, RH fermentation enriched Lactic acid, SCFAs and BCFAs concentrations, indicating that carbohydrate and protein were utilized. The results showed that Lactic acid and BCFA enrichments were comparable among volunteers. However, propionic, acetic and butyric acids as major SCFAs produced varied significantly among volunteers. The gut microbiota of V1, V2 and V3 used RH to produce more of propionic acid and less butyric acid, while V4 and V5 fermentation resulted in more of acetic and butyric acids. In addition, the abundance of *Bacteroides* and *Clostridium* in the microbiota of V1, V2 and V3 revealed strongly correlations with propionic and acetic acids. Whereas, in the microbial ecology of V4 and V5, the abundance of *Bacteroides*, *Clostridium*, *Bifidobacterium* showed significant correlation with the production of propionic, butyric and acetic acids. The results indicated that most of the LP could escape GI digestion and reach the colon to regulate the composition of gut microbiota and SCFAs of different individuals depending on their baseline microbiota.

## Methods

### Preparation of mushroom powder

Whole mushroom fruiting bodies were washed thoroughly to free them from mud, ferns and other extraneous material. White and gray fruiting bodies were dried in an oven at 60 °C and then pulverized into powder using a laboratory blender to produce *L. squarrosulus* powder (LP) that passed through a 40-mesh sieve.

### Nutritional value analysis of LP

Moisture, fat and ash content were determined by the procedure of AOAC method 925.45A, 922.06 and 920.153, respectively (AOAC 2016). Carbohydrate composition was measured according to In-house method TEC-CH-169 based on Compendium of Methods for Food Analysis, Thailand, 1st Edition, 2003. Protein was analyzed by In-house Method TE-CH-042 based on AOAC (2016) 981.10. Neutral detergent fiber (%NDF), Acid detergent fiber (%ADF), Acid detergent Lignin (%ADL), cellulose (%C), hemicellulose (%HC) and crude fiber (%CF) were obtained using the method by Van Soest, (1963). Energy was estimated using the following equation: Energy (kcal) = 4 (g protein) + 3.75 (g carbohydrate) + 9 (g fat).

### In vitro digestion

In vitro enzymatic hydrolysis of LP was performed using the method described by^24^. Mushroom powder was hydrolyzed sequentially with pepsin and pancreatic α-amylase (Sigma Chemicals Ltd.) by simulating gastric and small intestine conditions. Approximately 1 g LP was combined with 50 mL of HCl buffer (pH 1) containing; (g/L); NaCl, 8.00; NaH_2_PO_4_, 14.35; KCl, 0.20; CaCl_2_, 0.10; MgCl_2_.6H2O, 0.18; NaHPO_4_.2H_2_O, 8.25. The pH was adjusted using 5 M HCl and pepsin was added to obtained final concentration of 20 units per mL and incubated for 4 h. An aliquot, 5 mL was taken to new tube at 0 and 240 min. The pH was adjusted to 6.9 using 1 M NaOH following the addition of porcine pancreatic α- amylase solution to obtained final concentration of 1units/mL and further incubated for 6 h. An aliquot of 5 mL was taken to new tubes at 6 h. Total sugar and reducing sugar contents were determined using phenol sulfuric acid and DNS methods, respectively^56^. Percentage hydrolyzed (H%) was estimated by the ratio of reducing sugar released to total sugar content based on the equation below^57^. Protein content before and after digestion were also determined by Kjeldahl Method^58^.

$$\mathrm{Percentatge}\,\mathrm{hydrolysis} (\%)=\frac{\mathrm{Reducing}\,\mathrm{sugar}\,\mathrm{released} (\mathrm{final}- \mathrm{initial}\,\mathrm{sugar}) }{\mathrm{Total}\,\mathrm{sugar}\,\mathrm{content}-\mathrm{initial}\,\mathrm{reducing}\,\mathrm{sugar}}\times 100$$

The hydrolysates were transferred into a dialysis tube (3500MWCO, CelluSep Dialysis Membrane 5030–46, Seguin, USA), and dialyzed against water at 4 °C and then freeze-dried to obtain resistant hydrolysate (RH).

### Scanning electron microscope (SEM) analysis

The morphological characteristics of *L. squarrosulus* powder (LP) and the resistant hydrolysate (RH) were visualized. A High Resolution (HR) SEM (Quanta 200 FEG Instrument, Eindhoven, Netherlands) was used as previously reported^24^. Samples were placed on double adhesive tape fixed on a metallic stub, coated with gold and viewed at 1200 × resolution.

### Compositional changes determination

Spectro-microscopic measurements of carbohydrate, protein and fat fingerprint regions of LP and RH were achieved by a recently used procedure^20^. The measurements were made by synchrotron radiation (SR)-FTIR micro-spectroscopy (Hyperion 2000IR microscope coupled with VERTEX70 spectrometer, Bruker Optics, Germany) at beamline 4.1 at the Synchrotron Light Research Institute (SLRI), Nakhon Ratchasima 30,000, Thailand. The Samples were completely dried and grounded. Spectra collection were performed using OPUS8.0 software (Bruker Optics, Germany). Spectra acquisition were obtained with 36X objective lens using the transmission mode. All spectra were record from 4000 to 400 cm^−1^ with diamond compression cell at an aperture size of 20 × 20 µm; spectral resolution of 4 cm^−1^; 64 scans for background and sample.

### Probiotic bacteria fermentation

LP and RH were tested on selected probiotic bacteria; *Lactobacillus* ssp. (*L. reuteri* KUB-AC5 and *L. crispatus* JCM 5810), *Bifidobacterium* ssp. (*B. bifidum* KUB 2129 and *B. animalis* KUB 2194) following a reported method ^59^. *Lactobacillus* strains were cultivation in MRS medium at 37 °C under aerobic condition. Whereas, *Bifidobacterium* strains were grown in MRS-0.005 L-cysteine HCL broth at 37 °C anaerobically. Overnight cultures, adjusted to 0.5–0.6 OD absorbance at 600 nm were used to inoculate (1%) sterilized 10 mL appropriate broth containing 1% LP or RH or no sample (control). Enhanced activity (%) of *Lactobacillus* strains were evaluated at 4, 8 and 16 h, while Bifidobacterium strains were assessed at 6, 12 and 24 h using spread plating method on MRS agar plate.

$$\mathrm{Enhanced}\,\mathrm{activity} (\%)=\frac{\mathrm{SB}-\mathrm{CB}}{\mathrm{CB}}\times 100$$

where SB is the number of colonies (CFU/mL) of MRS medium with mushroom powder/hydrolysate and CB is the number of colonies (CFU/mL) from MRS medium without sample.

### Fecal inoculum and fermentation substrate

Fresh fecal samples were collected from five healthy volunteers (three females and two males aged from 26 to 31 years old) who had not taken antibiotics at least for 3 months. All volunteers provided written informed consent prior to specimen collection. Immediately following collection, each fecal sample was stored in an anaerobic culture swab and used within 2 h. The study was performed and approved in accordance with the guidelines of Kasetsart University Ethics Committee on Human Research. All methods in this study were in accordance with relevant guidelines and regulations of the Declaration of Kasetsart University Ethics Committee.

Fecal samples were homogenized with sterilized 0.1 M phosphate buffered saline, pH 7.0 to obtain 10% (w/v) suspension. The basal nutrient medium was prepared by adding 1.2 g peptone, 1.2 g yeast extract, 100 mg NaCl, 40 mg K_2_HPO_4_, 40 mg KH_2_PO_4_, 10 mg MgSO4, 10 mg CaCl_2_, 2 g NaHCO_3_, 25 mg hemin, 0.5 g cysteine-HCl, 0.5 g bile salt, 4 mL resazurin; 0.05 mg/mL stock, 2 mL Tween 80 and 20 μL vitamin K1 into 1 L distilled water and adjusted the pH to 7.0 by using 0.1 mol/L HCl solution. After the sterilization, the media was bubbled with CO_2_ to remove the O_2_ in solution and cool down to 3 7 ºC before use. This was performed in accordance with the reported method^60^ with minor modifications.

Water-jacketed fermenter containing basal medium was used as control group, and fermenter in other vessel supplemented with 1% resistant hydrolysate (RH) was used as treatment group. Then, the vessels were magnetically stirred with temperature control at 37 °C and pH maintained at pH 6.65–6.95. Anaerobic conditions were kept by sparing the vessels with oxygen-free nitrogen gas at 15 mL/min. The fermented samples for further analysis were collected at 0, 3, 6, 9, 12, 18 and 24 h fermentation.

### Detection of SCFA and BCFA metabolites

The samples collected during fermentation were centrifuged and the supernatants were filtered using a 0.22-μm filter unit (Millipore, Cork, Ireland). Twenty microliters were then injected into an HPLC system (Waters 1525 Binary HPLC, Milford Massachusetts, USA) equipped with a UV detector (Waters 2489 UV visible detector). An ion-exclusion Aminex HPX-87H column (300 × 7.80 mm; Bio-Rad, California, USA), set at 50 °C was employed. The mobile phase was 8 mM H_2_SO_4_ in HPLC-grade water, and the flow rate was 0.6 mL/min. Quantification of metabolites in the samples was carried out using calibration curves of lactate, acetate, propionate, iso-valerate, iso-butyrate and butyrate at concentrations of 1.0, 10, 20, 40, 80 and 100 mM. Tartaric acid (Sigma-Aldrich, UK) at a final concentration of 20 mM was used as the internal standard. The results were express as mmol/mL.

### DNA extraction

The bacterial DNA was isolated using QIAamp DNA Stool Mini Kit (Qiagen, Germany)^47^. The bacterial pellets were collected by centrifugation and transferred into 2 mL lysate tubes containing zirconium beads and inhibitEx was adding prior to agitation. The cell suspension was centrifuged and 400 µL of the supernatant was combined with 400 µL of buffer AL and, 17 µL of proteinase K in a new tube. The content was vortexed and incubated at 70 °C for 10 min, and allowed to cool down at room temperature. The lysate was added with 400 µL of ethanol and mixed by vortex. A 600 µL of the content was repeatedly applied to QIAamp spin column until all lysate was loaded. And then 500 µL of buffers, AW1 and AW2 were respectively added and centrifuged. After that 200 µL buffer ATE (elution buffer) was pipetted directly onto the QIAamp membrane. The DNA concentration was determined using a NanoDrop 1000 (NanoDrop Technologies, Wilmington, USA).

### Microbial analysis

The microbiota was analysed by MiSeq sequencing. DNA integrity was detected by the agarose gel electrophoresis. The genomic DNA was used as the template to amplify the V3–V4 regions of 16S rDNA with the forward primer (Illumina adapter sequence 1 + CCTACGGGNGGCWGCAG) and the reverse primer (Illumina adapter sequence 2 + GACTACHVGGGTATCTAATCC) and then specific index sequence was added. The PCR products were purified for library construction. High throughput sequencing was performed on the Illumina MiSeq platform (Illumina, USA) to generate 2 × 250 bp paired-end reads. The reverse reads were right truncated from the right to 240 bps as a quality control measure. After that the primers at the 5’ of reads were trimmed using seqtk (https://github.com/lh3/seqtk). The processed pair of sequences were denoised, then merged into amplicon sequence variants (ASVs) using DADA2 pipeline (v.1.10) with pseudo-pooling mode. We assigned a taxonomy label to each ASV with QIIME2’s sklearn classifier (v.2020.2) and Greengenes (v.13.8)^61^. We exclude ASV that either has no phylum label and a singleton from the analysis. Alpha diversity and Beta diversity were computed with vegan (v.2.5–6)^62^ and GUniFrac (v.1.1)^63^.

### Statistical analysis

Statistical testing was performed on two groups; control (C) and treatment (S/RH) of five fecal samples taken at six periods (0, 3, 6, 9, 12 and 24 h) for metabolites (SCFA and BCFA) concentrations, and at four periods (0, 6, 12, 24 h) for bacterial populations. Differences between bacterial groups were computed using T-test and among individuals were achieved using one-way ANOVA for continuous data in SPSS. Shannon, Simpson and S. chao 1 index were used to estimate the community diversity. A Bray–Curtis and Unifrac similarity matrix was derived and a permutational analysis of variance (PERMANOVA) pair wise comparison was conducted to compare all community samples. A *p*-value of < 0.05 was considered statistically significant. A heat map summarizing Spearman correlations between metabolites, bacterial groups and RH treatment at various time points of fermentation was determined using R.
